# Real-Time CO_2_ Production Monitoring in Stored Oats as an Indicator of Type A Trichothecenes and Ochratoxin A Contamination Under Simulated Environmental Conditions

**DOI:** 10.3390/toxins17030132

**Published:** 2025-03-11

**Authors:** Abimbola Oluwakayode, Michael Sulyok, Franz Berthiller, Carol Verheecke-Vaessen, Rudolf Krska, Angel Medina

**Affiliations:** 1 Magan Centre of Applied Mycology, Cranfield University, College Rd. Wharley End, Bedford MK43 0AL, UK; abimbola.oluwakayode@cranfield.ac.uk (A.O.); c.verheecke@cranfield.ac.uk (C.V.-V.); 2 Institute of Bioanalytics and Agro-Metabolomics, Department of Agricultural Sciences, BOKU University, Konrad-Lorenz, Str. 20, 3430 Tulln, Austria; michael.sulyok@boku.ac.at (M.S.); franz.berthiller@boku.ac.at (F.B.); rudolf.krska@boku.ac.at (R.K.); 3 Institute for Global Food Security, National Measurement Laboratory, Centre of Excellence in Agriculture and Food Integrity, Queen’s University Belfast, 19 Chlorine Gardens, Belfast BT9 5DL, UK

**Keywords:** *Fusarium langsethiae*, T-2/HT-2 toxin, ochratoxin A, carbon dioxide respiration rate, water activity, LC-MS/MS

## Abstract

Grain industries are interested in an integrated approach to in-silo grain quality and safety management using carbon dioxide (CO_2_) measurement with temperature and moisture monitoring. Our study investigates if CO_2_ production could predict mycotoxin production (T-2 toxin, HT-2 toxin, its glucoside, and ochratoxin A (OTA)) and identify storage conditions exceeding legislative limits in stored oats for the first time. The influence of water activity (a_w_) levels (0.70–0.95 a_w_), temperature (15 and 20 °C), and storage duration on (a) *Fusarium* populations, (b) CO_2_ respiration rates (RRs), and (c) mycotoxin concentrations in stored oats was examined. One hundred and twenty samples were analysed for multiple mycotoxins by LC-MS/MS. Substantial differences were found in the RRs of oats at ≥0.90 a_w_ at both temperatures. A moderate positive correlation between CO_2_ and mycotoxins was noticed and mycotoxins exceeded their limits at ≥0.90 a_w_ (22% moisture content) when RR ≥ 25 µg CO_2_ kg^−1^ h^−1^. This knowledge forms the basis for developing decision support systems for improving oats’ storage management.

## 1. Introduction

Oats (*Avena sativa* L.) are significant cereal crops highly valued for their dietary benefits and growing market demand with the global oat market projected to reach USD 10.8 billion by 2032 [1]. It is well suited to cooler environments, requiring an optimal temperature range of 15–25 °C and moist conditions [2]. However, post-harvest losses due to spoilage, primarily caused by moulds and consecutive mycotoxin contamination, remain a significant challenge affecting around 14% of food produced globally [3]. Mycotoxins are toxic compounds produced by certain fungi under specific environmental conditions. The accumulation of mycotoxins in oats poses both economic concerns and health risks [4].

Fungal species such as *Fusarium, Penicillium*, and *Aspergillus* are commonly linked to oat spoilage and diseases, including *Fusarium* head blight [5,6,7,8,9]. These moulds thrive in warm and humid storage conditions and reduce the quality and quantity of grains by producing harmful mycotoxins. *Fusarium langsethiae* is an important *Fusarium* species found in oats [10] producing type A trichothecenes (T-2/HT-2 toxins). Other fungal genera produce ochratoxin A (OTA), deoxynivalenol (DON), and fumonisins [11,12].

Plants can metabolise T-2 and HT-2 produced by *Fusarium* species to modified forms such as HT-2-glucoside or T-2-glucoside, among others [13]. These modified forms are considered less toxic but can be converted back to their native form to regain their toxicity in the mammalian digestive tract [14].

To minimise the risks of mycotoxins, various countries and international organisations have established regulations and guidelines to control mycotoxin levels in food and feed. These regulations aim to ensure food safety and protect consumer health by setting maximum permissible levels of mycotoxins in various commodities. In the European Union, the maximum level for the sum of T-2 + HT-2 in unprocessed oats with inedible husks was enforced in 2024 and is 1250 μg/kg [15].

Respiration in stored cereal grains is the metabolic process of breaking down stored carbohydrates, such as starch, into simpler compounds like carbon dioxide (CO₂) and water, releasing energy [16]. This process is influenced by abiotic components (such as moisture and temperature), oxygen availability, and grain condition in storage [17]. Therefore, CO_2_ produced in a stored grain mass is due to the respiration of the grains and the biotic factors present such as microorganisms and insects. Many studies have discussed the impact of abiotic factors on the growth and mycotoxin production of *Fusarium* species [18,19,20].

As the moisture content (MC) rises in the grain silo, so does the temperature. The temperature of the external environment affects the silo wall, leading to condensation within the silo when the temperature decreases. This, in turn, increases the moisture content in the grains, causing them to respire and elevating the temperature inside the silo, ultimately resulting in grain spoilage. The movement of moisture and CO_2_ concentrations within a bulk grain mass was described by Ramachandran et al. [21].

Due to the inter-relationship between MC, temperature, and gas produced in grain silos, it is important to adopt an integrated approach that combines the monitoring of moisture, temperature, and CO_2_ concentrations in stored grain to improve grain quality and safety in storage. Some studies have employed this approach [22,23,24,25]. However, these studies did not consider the relationship between these abiotic factors (MC × temperature × CO_2_) and mycotoxins produced in the grains. Mylona et al. [26] assessed fungal growth and dry matter loss (DML) with the amount of CO_2_ produced in stored irradiated wheat and maize under different interacting conditions of water activity (a_w_) and temperature. They reported increased respiration rates (RRs) as a_w_ and temperature increased with varying production patterns of *Fusarium* species. However, gas measurements in the irradiated grains were conducted by gas chromatography (GC). This does not accurately reflect the real-time CO_2_ measurements of the natural mycobiota of non-irradiated grains. Garcia-Cela et al. [27] also reported that RRs and related DMLs can indicate the relative likelihood of mycotoxin contamination in natural and irradiated wheat grains. The RRs were also measured with GC, and the grain size (10 g) is considerably smaller compared to the study of Oluwakayode et al. [28]. They investigated the relationship between CO_2_ measurement with real-time CO_2_ sensors and ochratoxin concentrations produced in a mini silo of wheat grains under different a_w_ and temperature conditions, and identified that RR increased as a_w_ increased and OTA concentrations were highest at the wettest a_w_ level (0.95 a_w_). They highlighted a positive correlation between the CO_2_ produced and OTA produced at the wettest storage conditions.

This study aims to investigate the relationship between the respiration rates, type A trichothecenes, and OTA concentrations in mini silos of oats under different interacting environmental factors. We wanted to determine if CO_2_ production could indicate the risk of these mycotoxin contaminations above legislative limits in stored oats.

The objectives are to investigate the effect of storage conditions (0.70, 0.90, and 0.95 a_w_ at 15 and 20 °C) on (a) mycotoxigenic fungal populations, (b) CO_2_ RRs, and (c) the sum of T-2/HT-2 toxin + HT-2-glucoside and OTA produced in naturally contaminated oats, and (d) to examine the relationship between the CO_2_ and mycotoxins concentrations in contaminated oats.

## 2. Results

### 2.1. Fungal Populations and Isolations in Contaminated Oats

Table 1 and Table 2 show the mean values of the colony-forming units (CFUs) and the frequency of isolation of potentially mycotoxigenic fungal genera in oat treatments per media.

*Fusarium* populations were observed at 0.70 and 0.90 a_w_, while *Penicillium* populations were mostly present at all storage conditions (Table 1).

*Aspergillus* sect. *Nigri* and *Aspergillus* sect. *Flavi* were only present at 0.95 a_w_ in the contaminated oats at 20 °C. *A*. sect. *Nigri*’s mean values are 5.9 ± 6.1 and 5.9 ± 6.0 on MEA+ and DG18+, respectively, while *A*. sect. *Flavi*’s mean values are 5.1 ± 5.3 and 5.2 ± 5.4 on MEA+ and DG18+, respectively.

Pictorial representations of fungal populations or isolations in the contaminated oats on MEA+ media at all a_w_ levels at 15 °C are shown in Figure S1 in the Supplementary Materials.

*Fusarium* isolation was higher in DG18+ media than in MEA+ at 0.70 and 0.90 a_w_ but absent in the 0.95 a_w_ on DG18+ media. Of the potentially mycotoxigenic fungal strain, *Penicillium* was relatively abundant in the oats in both media and highest at 0.95 a_w_. Interestingly, *Aspergillus* species were relatively low under drier storage conditions but present at 0.95 a_w_, especially at 20 °C where *Fusarium* growth was least observed.

### 2.2. The Influence of Storage Conditions on Respiration Rates in Contaminated Oats

**Figure 1:**
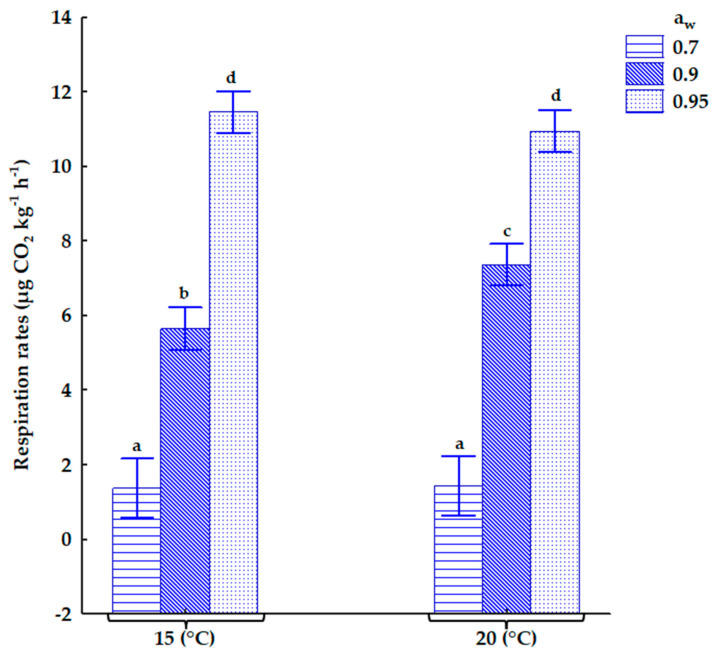
The effect of the interactions of a_w_ × temperature on the respiration rates in contaminated oats. Using the Tukey HSD test, different letters show significant differences in respiration rates. a_w_—water activity.

The RR increased significantly as the a_w_ was increased in the stored oats (Figure 1). However, there were no significant differences in RRs between both temperatures for each a_w_ except at 0.90 a_w_. An increase in storage days either as a single factor or its interactions with other conditions does not significantly impact RR. The interactions of a_w_ × temperature significantly impact the RR in the stored oats as shown in Figure 1.

The mean values of RRs (µg CO_2_ kg^−1^ h^−1^) in contaminated oats at all storage conditions are shown in Table 3. The RR was more than four times higher under wetter conditions than at 0.70 a_w_ at both temperatures.

Figure S2 in the Supplementary Materials shows the respiration rates produced at day 0, day 10, and day 20 for each a_w_ and temperature.

### 2.3. Method Validation Performance and the Influence of Storage Conditions on Mycotoxin Concentrations in Contaminated Oats

#### 2.3.1. Method Validation Performance in the Oat Matrix

The extraction efficiency (RE) of each analyte was within the acceptable range (70–120%) according to the amended guideline set by European Commission regulation No. 2021/808/EC [29]. The goodness of fit of the calibration curve for each analyte was acceptable, with r^2^ values (coefficient of determination) of >0.990. The relative standard deviation (RSD %) was satisfactory for the validated analytes <14% except for OTA (17%). The relative standard deviation of the within-laboratory reproducibility (RSD_WLR_), the matrix effect or the signal suppression/enhancement (SSE), and the apparent recovery (R_A_ %) were calculated from the average of 48 replicates of the three different lots of oats spiked in quadruplicate across three separate days. The limit of detection (LOD) and limit of quantitation (LOQ) for the analytes ranged from 0.9–4.1 µg/kg and 2.9–13.6 µg/kg, respectively, which were lower than the minimum acceptable levels for the regulated mycotoxins in unprocessed oats [15,30]. The mean values of the R_A_, RE, RSD, LOD, and LOQ for each analyte are shown in Table 4.

#### 2.3.2. The Influence of Storage Conditions on Mycotoxin Concentrations in the Naturally Contaminated Oats

In the naturally contaminated oats, the concentrations of the sum of T-2 + HT-2 including HT-2 glucoside were below the maximum limit (1250 μg/kg) at all storage conditions except at 0.90 and 0.95 a_w_ at 20 °C on day 20 (Table 5). OTA levels were highest at 0.95 a_w_ for both temperatures and increased significantly by 12% as a_w_ rose from 0.90 to 0.95 a_w_.

**Figure 2:**
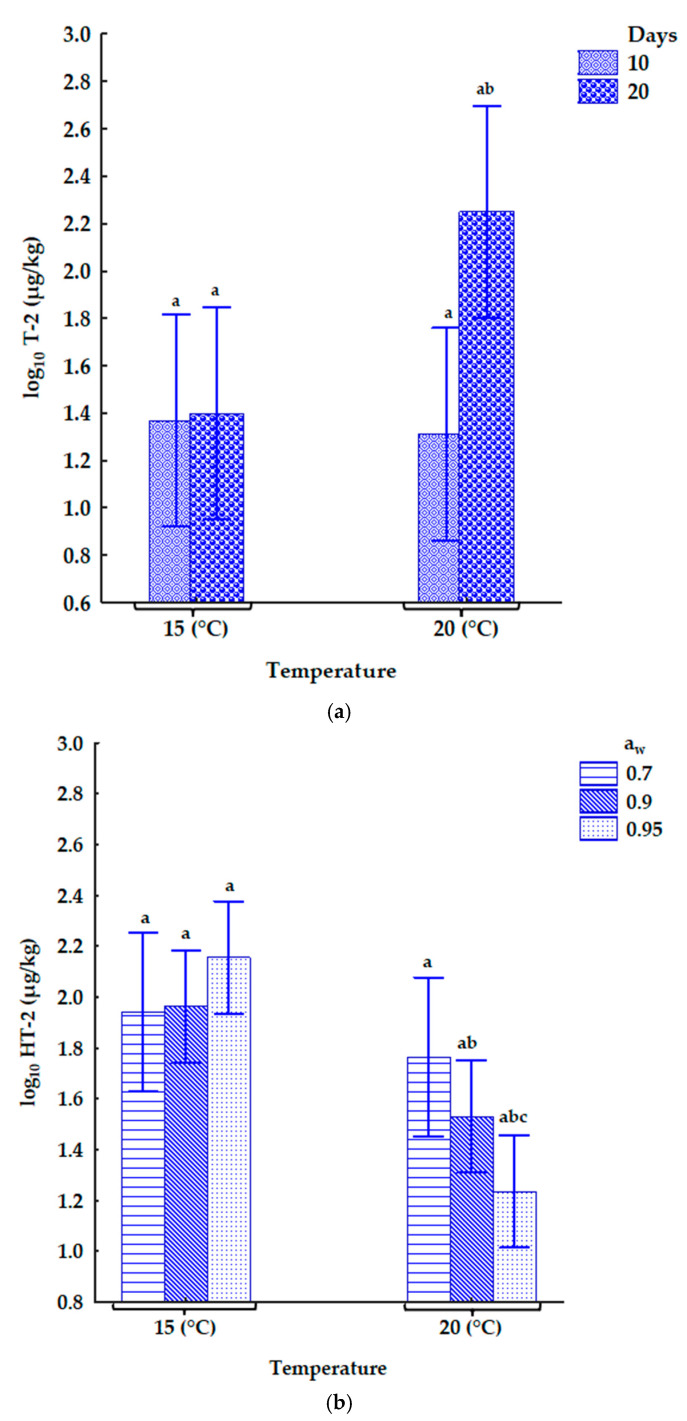

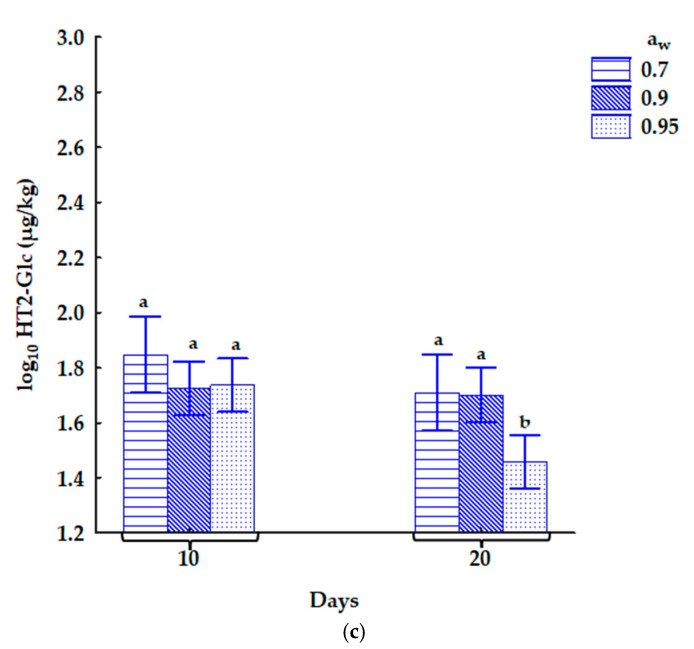
The effect of the interactions of (**a**) storage days × temperature on T-2 toxin, (**b**) a_w_ × temperature on HT-2 toxin, and (**c**) a_w_ × storage days on HT2-Glc. Using the Tukey HSD test, the same letters show no significant differences in type A trichothecenes concentrations. The vertical bar denotes 95% confidence intervals. a_w_—water activity.

The influence of the interactions of storage conditions on type A trichothecenes and OTA production in naturally contaminated oats is shown in Figure 2 below.

In Figure 2a, the Tukey HSD test shows that storage days significantly impacted T-2 concentrations (*p* = 0.0416) at 20 °C. a_w_ as a single factor or its interaction with other storage conditions did not significantly impact T-2 concentrations.

HT-2 concentrations decreased significantly as temperature increased at 0.95 a_w_. However, HT-2 concentrations did not vary significantly across all a_w_ levels at 15 °C (Figure 2b). Temperature and the interaction of a_w_ × temperature significantly impacted HT-2 concentrations. Its concentrations decreased significantly as temperatures increased at 0.90 and 0.95 a_w_.

Figure 2c shows the interaction between a_w_ × days on HT-2-Glc concentrations. There were no significant differences in its concentrations at each a_w_ level on day 10. However, the concentrations of HT-2-Glc decreased significantly at 0.95 a_w_ on day 20 (Figure 2c). Water activity, temperature, and storage days as single factors significantly impacted HT-2-Glc concentrations. Its concentrations in the contaminated oats increased as these factors increased.

##### The Influence of Storage Conditions on OTA Concentrations in the Naturally Contaminated Oats

**Figure 3:**
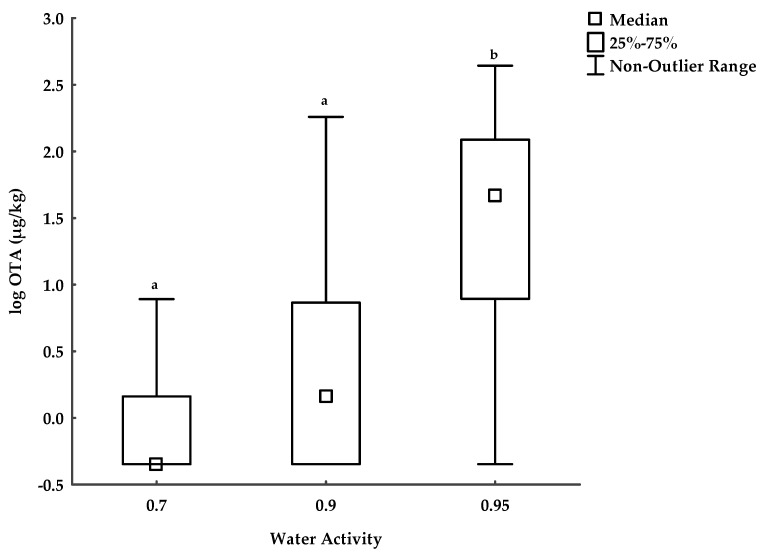
The effect of a_w_ on OTA concentrations in contaminated oats. Different letters show significant differences in OTA concentrations among a_w_ levels.

In Figure 3, the mean of the OTA concentrations was not significantly different between 0.70 and 0.90 a_w_. However, OTA concentrations significantly increased as a_w_ reached 0.95 a_w_. Moreover, an increase in storage days significantly affected OTA concentrations only at 0.95 a_w_ (see Table 5).

#### 2.3.3. Relationship Between Respiration Rates (RR) and Type A Trichothecenes and Ochratoxins in Oats

**Figure 4:**
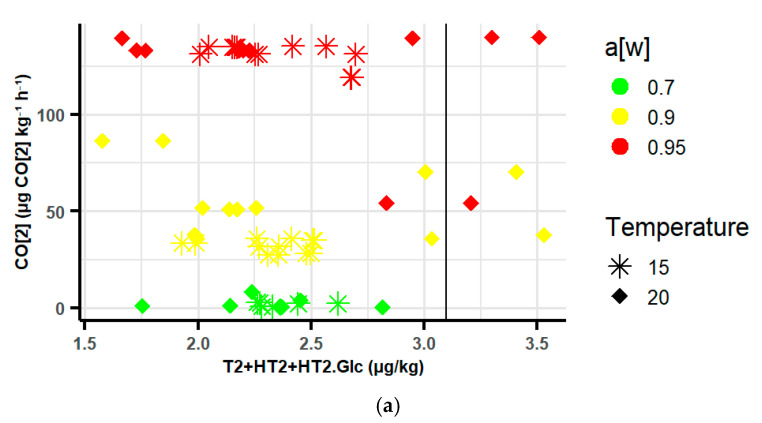

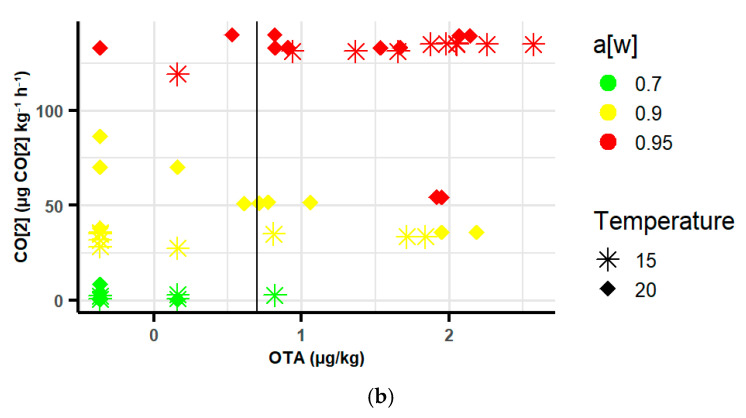
The relationship between the RR and the concentrations of (**a**) the sum of T-2 + HT-2 + HT-2-Glc and (**b**) OTA in the contaminated oats at all storage conditions. a[w]—water activity. Vertical lines indicate maximum levels of 1250 μg/kg for T-2 + HT-2 and 5 μg/kg for OTA concentrations. Temperature unit: °C.

At 0.70 a_w_, the RR was low, and the sum of the concentrations of T-2 + HT-2 + HT-2-Glc in the naturally contaminated oats at 15 °C did not exceed the maximum limit. However, as a_w_ and temperature increased, the RR increased with a corresponding increase in T-2 + HT-2 + HT-2-Glc concentrations. It exceeded the maximum limit (1250 µg/kg) at ≥0.90 a_w_ and ≥27 µg CO_2_ kg^−1^ h^−1^ as indicated by the reference line in Figure 4a.

Similarly, OTA concentrations increased as the CO_2_ RR increased and mostly exceeded the maximum limit at 0.90 a_w_ and ≥27 µg CO_2_ kg^−1^ h^−1^ in the contaminated oats with an exception at 0.70 a_w_ and 15 °C (Figure 4b).

The relationship between CO_2_ RR, type A trichothecenes, and ochratoxins in the contaminated oats was examined with linear regression and a generalised linear model (GLM). The adjusted R^2^ value for the OTA model is 0.3832. This suggests a moderate positive relationship as CO_2_ and storage days were significant predictors (*p* < 0.05) of ochratoxin concentrations. The GLM model showed similar results. The Pearson correlation coefficient (r = 0.5322) shows a significant correlation between CO_2_ and OTA concentrations (*p*-value < 0.0001) in the contaminated oats.

The adjusted R^2^ value for the T-2 + HT2 + HT2-Glc model was 0.06013, with storage days as a significant predictor. The Pearson correlation coefficient (r = 0.0039) shows an insignificant correlation between CO_2_ and the sum of T-2 + HT2 + HT2-Glc (*p*-value = 0.976) Table 6 below.

The model’s accuracy was assessed through cross-validation by comparing predicted mycotoxin values with known ones (Figures S3 and S4 in Supplementary Materials), but the linearity of the plots was not satisfactory. These models alone cannot accurately predict OTA and T-2 + HT2 + HT2-Glc concentrations using only CO_2_ levels as a predictor, as other storage conditions such as a_w_ and temperature influence CO_2_ and mycotoxin levels in stored oats.

## 3. Discussion

To the best of our knowledge, the study of the relationship between the real-time CO_2_ measurement and the risk of trichothecenes and OTA in oats using a mini-silo pilot study has not been well documented. This study measures the real-time RR in stored oats with integrated CO_2_ sensors to examine the risk of type A trichothecenes and ochratoxins contamination under storage conditions 0.70, 0.90, and 0.95 a_w_, and 15–20 °C. These a_w_ levels correspond to the oat moisture content (MC) of 14%, 22%, and 25% on a wet-weight basis [32]. These MCs can be achieved in stored oat grains due to inefficient drying or compromised storage conditions resulting from silo leaks, pest infestations, seasonal rainfall, or temperature changes [33].

Fusarium is commonly known to thrive in wet and humid conditions. Surprisingly, its populations and isolations at 0.95 a_w_ were relatively low in Table 1 and Table 2. At the same a_w_ level, Penicillium had the highest fungal isolations (100%) at 15 °C, indicating that it is a fungus adapted to temperate climates, while Aspergillus isolations were highest at 20 °C. Medina and Magan [18] reported the growth limit of *F. langsethiae* strains from the UK and northern Europe on an oat-based media at 0.92–0.93 a_w_ at 10 °C. No growth was recorded at 0.90 a_w_ regardless of strain type and temperature studied. A similar study on an oat-based medium, reported no growth rate for the *F. langsethiae* strain at 0.90 a_w_; however, growth was recorded at 0.95 a_w_ at 15 and 20 °C after 10 days of incubation with a growth limit at 0.93 a_w_ [34]. In contrast to these two studies, our findings show the populations of Fusarium in situ at 0.70 and 0.90 a_w_ with low or no populations at 0.95 a_w_. This could be due to the adaptability or resilience of the spores in response to stress from drier conditions [35,36].

### 3.1. Respiration Rates in Contaminated Oats

Respiration rates have been an important factor indicating the deterioration of cereal grains in storage. Grains with fungal infections have higher respiration rates because of the increased metabolic activity associated with the infection [37]. Over many decades, research has highlighted that high moisture content leads to high RRs in stored grains, while grains stored at a drier a_w_ of 0.70 a_w_ (≤14% MC equivalent), produced a negligible amount of RRs [38,39,40]. Recent studies have supported this [23,27,28]. Our results show a marked increase in RR with a rise in a_w_ in the oats. The RR was more than 100% higher at ≥0.90 a_w_ for both temperatures. This result aligns with a recent study by Oluwakayode et al. [28] which investigated the impact of a_w_ on RR in stored wheat. They reported a rapid increase in RR at a moisture content > 16% (0.80 a_w_) within the first 10 days of storage in a mini silo of wheat. The elevated level of CO_2_ could arise from pockets of grain mass within the silo due to a combination of factors, such as moisture content, the presence of moulds or other microbes, insect infestation, and temperature [22].

Our findings showed that an increase in temperature alone did not significantly increase the RR, but its interactions with other storage conditions did. An important observation is that the CO_2_ produced was highest at 0.95 a_w_ for both temperatures. However, it did not correspond to Fusarium growth at the same a_w_ level. The CO_2_ at each a_w_ was produced by both the grain and the fungal communities which include both spoilage and toxigenic moulds within the oats in the silo. It is therefore unrealistic to separate the grain’s respiration from the fungus’s respiration during CO_2_ monitoring. As CO_2_ increased and become saturated within the silo, the CO_2_ sensor’s concentration limit (50,000 ppm) was reached, and the readings remained constant throughout the storage. Hence, for both temperatures, the wettest water activity (0.95 a_w_) showed the same real-time respiration rates from day 10 until day 20 without any significant difference.

Despite observing low or no Fusarium growth from the fungal population and isolation results at 0.95 a_w_ for both temperatures, considerable levels of type A trichothecenes were found in the oats samples with T-2 concentrations alone exceeding the maximum limit of the sum of T-2 + HT-2 (1250 µg/kg) in most cases. It is understood that Fusarium’s growth may be suppressed under low-oxygen and high-humidity conditions, prompting it to produce mycotoxins such as fumonisins, zearalenone, and trichothecenes to deter microbial competition and adapt to stress [41]. Fusarium regulates growth and toxin synthesis through separate metabolic pathways, allowing toxin production independent of growth [42].

### 3.2. Type A Trichothecenes in Naturally Contaminated Oats

In the contaminated oats, T-2 toxin levels were at least 1.4 times lower as a_w_ increased. However, on day 20 at 20 °C, T-2 concentrations were 6 times higher as a_w_ increased from 0.70 a_w_ to 0.90 a_w_ and with a 48% increase from 0.90 to 0.95 a_w_. Under low and medium conditions, the concentrations of HT-2-Glc were higher than those of T-2. However, in the wettest conditions with high levels of T-2, this pattern was reversed, suggesting that the plants may have been overwhelmed by the toxin, preventing them from detoxifying it effectively. The significant levels of HT-2-Glc highlight the necessity of measuring it to ensure accurate assessments of the total mycotoxin content in grains.

Additionally, HT-2 concentrations were more than twice significantly higher than T-2 concentrations in wet conditions (0.90–0.95 a_w_) at 15 °C. Some studies have reported higher levels of HT-2 than T-2 toxins in oats, although without considering the impact of different environmental factors [43,44]. The higher concentrations of HT-2 than T-2 could be due to the deacetylation of T-2 to HT-2 by oat grains or the rapid hydrolysis of T-2 to HT-2 by the natural mycobiota in response to ecological stress. Further investigation is needed to understand the metabolic pathways of HT-2 in *F. langsethiae* [45].

In contaminated oats, it is expected that the concentrations of type A trichothecenes will increase under wet conditions, as most Fusarium species thrive in such environments. However, *F. langsethiae* has been noted to have slow colonization rates, having an inability to compete with other phyllosphere Fusarium species, such as *F. poae*, in colonizing oats [11]. Kahla et al. [46] reported variations in the effect of the acclimatisation of a range of *F. langsethiae* strains on growth rates and toxin production under ambient (400 ppm) and elevated (1000 ppm) CO_2_ on oat-based media. Further research is necessary to clarify the interactions of *F. langsethiae* with other mycobiota, and the implications of such competition for dominance in the oat phyllosphere and its influence on toxin production.

### 3.3. OTA in Naturally Contaminated Oats

Ochratoxin A is generally considered a storage toxin produced mostly by Penicillium or Aspergillus species. OTA increased with temperature rise except at 0.95 a_w_ on day 20 in the contaminated oats. The highest OTA level was at 15 °C and 0.95 a_w_, exceeding the maximum limit (5 µg/kg). This can be linked to the high prevalence of Penicillium populations or isolations in the oats at 0.95 a_w_ and 15 °C where Fusarium populations were least observed. However, the concentrations of OTA were lower than those of type A trichothecenes under these storage conditions. A similar study reported OTA production to be highest at 0.95 a_w_ and 15 °C in stored wheat grains [28], supporting that Penicillium thrives well in temperate conditions. Cairns-Fuller et al. [47] reported that *P. verrucosum* growth and OTA production can occur at ≥0.85 a_w_ in wheat grains. Our findings observed measurable values of OTA ≥ 0.90 a_w_. Other studies have reported high OTA levels at ≥0.90 a_w_ [48,49,50]. It is important to stress that the effect of the interaction of a_w_ × storage days significantly increased OTA concentrations in the stored oats. Similar to our findings, Dhungana et al. [50] reported a significant impact of the interaction of the a_w_ × incubation period on OTA accumulation. The highest OTA level was at 0.90 a_w_ and it increased with a prolonged incubation period.

### 3.4. Relationship Between CO_2_ and Mycotoxins in Naturally Contaminated Oats

The sum of type A trichothecenes and ochratoxins concentrations were not strongly correlated with RR in the stored oats. This is because the RR was produced by the entire fungal community and the oat grains, not only by Fusarium and Penicillium species. Moreover, a direct correlation of RR with these mycotoxins may not be feasible as other storage conditions influence changes in the RR and the mycotoxins produced. Our findings suggest that at ≥0.90 a_w_ and an RR of ≥25 µg CO_2_ kg^−1^ h^−1^, the sum of type A trichothecenes and ochratoxins will exceed their legislative maximum limits in stored oats. Therefore, monitoring environmental factors during oat storage is important to minimise mould growth and mycotoxin contamination.

### 3.5. Conclusions

This study shows the behaviour of *F. langsethiae* within a natural mycobiota in stored oats, producing high levels of T-2 toxins under the wettest condition of 0.95 a_w_ despite appearing to be outgrown by Penicillium species. Water activity and storage days significantly affected RR and the concentrations of type A trichothecenes and OTA. The linear regression model shows a moderate positive relationship between the CO_2_ RR and OTA concentrations. Mycotoxin exceeded legislative limits at ≥25 µg CO_2_ kg^−1^ h^−1^ at ≥0.90 a_w_. These findings support the potential use of CO_2_ RR as an indication of the relative risks of oats’ contamination by these mycotoxins and present preliminary results that can be integrated with field and agronomic data to develop decision support systems for improving oats’ storage management. Further experiments in larger scale storage facilities that consider additional parameters (e.g., shape and size of silos, position of the sensors to capture CO_2_ production) could provide more “at scale” results to support industrial uptake of these monitoring systems.

## 4. Materials and Methodology

### 4.1. Oat Grains and Moisture Adsorption Curve Analysis

Mascani oats harvested in England (2022 harvest) were stored at 4 °C for 2 weeks before the experiment. AquaLab water activity meter 4TE Decagon devices, Inc. (Pullman, WA, USA) were used to analyse the initial a_w_ of grains. The grains’ moisture content (MC) was analysed by drying grains in the oven at 105 °C overnight. The MC values were calculated on a dry weight basis. The moisture adsorption curve was adapted from Oluwakayode et al. [16]. The relationship between the MC (dry weight basis) and a_w_ values was plotted and noted (Figure S5 in Supplementary Data).

### 4.2. Mini-Silo CO_2_ Sensing Storage Experiment

Targeted water activities (0.70, 0.90, 0.95 a_w_) were achieved in 2.5 kg of oat grains weighed into a 12-litre box mixed with a known amount of water. The grains were vigorously mixed and stored at 4 °C overnight. Grains were then equilibrated at the targeted temperature (15 and 20 °C) in rooms for 3 h, and the equilibrated grains’ a_w_ values were measured. Grains were transferred into a coffee flask with a centrally placed ATEX-compliant sensor probe. Gaseous movement in and out of the flask was minimised by placing cotton wool in the headspace of the flasks. There were three flasks (replicates) for each a_w_ level and the nine flasks were stored at each temperature condition. The probe is made of CO_2_, temperature, and relative humidity sensors connected to a computer system where their respective real-time monitoring readings from the stored grains were collected for 20 days. In total, 60 g of the contaminated samples was collected on days 10 and 20 from each flask with a sterile probe into sterile jars. Multiple small sizes of grains were taken from different spots to have a good representation of the bulk sample. The a_w_ of the grains was monitored before and after sampling. In total, 20 g of the sub-sample grains was analysed for fungal populations and isolations, while the remaining 40 g was oven dried at 50–55 °C overnight, ground, and stored at −20 °C before further analysis. The CO_2_ sensors were calibrated before each experiment for each treatment and temperature studied. After sampling on day 10, the grain inside the flask is 60 g less; the new weight is considered for calculating the respiration rates for day 20. The respiration rate was calculated using the diffusion formula in Equation (1) adapted from Raudienė et al. [27].

(1) $$Respiration\;rates=\frac{∆CO2\times MCO2\times Vh}{Vm\times m\times ∆t}$$

where ΔCO_2_ is the change in CO_2_ volumetric concentration in ppm, MCO_2_ is the molar mass of CO_2_ gas = 44.01 (gmol^−^^1^), V_h_ is the volume of headspace in the jar (L), V_m_ is the molar volume of a gas (Lmol^−1^), m is the mass of the oat sample (kg), and Δt is the duration (h) of ΔCO_2_.

The molar volume was determined by analysing the storage temperatures.

$$Vm=\frac{R\times T}{P}$$

where R is the gas constant (Jmol^−^^1^ K^−^^1^), T is temperature (K), and P is pressure (atm)

### 4.3. Fungal Populations and Isolations

According to the manufacturer’s instructions, 15 g of peptone agar was mixed in one litre of distilled water. A total of 9 mL was dispensed into 25 mL universal glass bottles, autoclaved at 121 °C for 15 min, and allowed to cool.

One gram of contaminated oat kernels was soaked in sterile 9 mL peptone water in a 25 mL universal bottle for 3 h. One ml of this bottle was serially diluted (by ten-fold dilution) to the 0.0001 M solution bottle. Sterile tips were used between each dilution and the universal bottle’s content was shaken using a vortex. 100 µL of the dilution aliquots was pipetted and reversely plated onto Dichloran Glycerol 18 agar (DG18+: Oxoid CM0729) and Malt Extract Agar (MEA+: Oxoid CM59) media in 3 replicates and spread out with a sterile metal spreader. The plates were incubated at 25 °C for 7 days. Fungi genera were identified visually with a stereoscope (Olympus 308136, Wild M7A, Heerbrugg, Switzerland) based on morphological characteristics [51]. Colonies were counted with a colony counter (Gallenkamp, Cambridge, UK) and expressed as log_10_ CFUs/g-dry weight.

### 4.4. Fungal Isolations

A total of 25 grains were directly inoculated on DG18+ and MEA + media to identify the dominant fungal genera in the contaminated grains. Five grains were placed equidistantly on each medium plate of five replicates and incubated at 25 °C for 7 days. Fungal genera were identified visually with a stereoscope based on morphological characteristics. The frequency of isolation was calculated using Equation (2) below.

(2) $$\%\;Frequency\;of\;isolation=\frac{Total\;contaminated\;grains}{Total\;grains\;in\;plates\;(25\;grains)}\times 100$$

### 4.5. Mycotoxin Analysis

#### 4.5.1. Chemical Reagents

T-2 toxin, HT-2 toxin, and ochratoxin A standard solutions were purchased from Romer Labs (Tulln, Austria), and HT-2-toxin-3-O-β-D-glucoside was synthesized by Michlmayr et al. [52]. LC-MS/MS-grade methanol, acetonitrile (ACN), and formic acid (Honeywell, Seelze, Germany); ammonium acetate (MS grade, Sigma-Aldrich, Darmstadt, Germany); and glacial acetic acid (HAc, Sigma-Aldrich, Burlington, MA, USA) were used. Water was purified successively by reverse osmosis and using a Pure LAB water system (VEOLIA, High Wycombe, UK).

#### 4.5.2. LC-MS/MS

The liquid chromatography–tandem mass spectrometry (LC-MS/MS) machine used in this study was an LQTRAP 5500+ MS/MS system (SCIEX) equipped with a Turbo V electrospray ionization (ESI) source, coupled to an ExionLC AD System (SCIEX, Framingham, MA, USA). Chromatographic separation was performed at 27 °C on a Gemini C18 Column, 100 × 4.6 mm (Phenomenex, Torrance, CA, USA).

LC-MS/MS was performed in the time-scheduled MRM mode in positive and negative polarities in one chromatographic run per sample by scanning two fragmentation reactions per analyte. Elution was carried out in binary gradient mode. Both mobile phases contained 5 mM of ammonium acetate and were composed of water/methanol/acetic acid at 89:10:1 (*v*/*v*/*v*; eluent A) and 2:97:1 (*v*/*v*/*v*; eluent B), respectively, with a sample injection volume set at 5 µL, with a total runtime of 21 min. The gradient elution program for the elution of mycotoxins was as follows: 0 min 5% B, 0.5 min 5% B, 2.5 min 70% B, 3.5 min 95% B, 5 min 95% B, and 7.5 min 5% B. The mass spectrometry parameters used are outlined in Table 7.

#### 4.5.3. Method Validation

The acceptable performance criteria of analytical methods set and updated by European Commission regulation No. 2021/808/EC [29] were used to validate the optimised LC-MS/MS method for mycotoxin analysis in the oat samples. The performance characteristics evaluated were linearity (r^2^), limit of detection (LOD), limit of quantification (LOQ), matrix effect or the signal suppression/enhancement (SSE), recovery of the extraction process (RE), absolute recovery (R_A_), and repeatability.

Working standard solutions were prepared to contain 25 µL each of stock solutions of T-2 toxin, HT-2 toxin, HT-2-Glucoside, and Ochratoxin A mixed in an ACN solution. In total, 50 mg of three lots of homogenised oat samples was spiked at the working solution concentration and a 10-fold dilution level. This was performed in duplicate. Spiked samples were placed in the dark overnight for solvent evaporation and analyte–matrix interaction. The spiked and unspiked samples were extracted with ACN:H_2_O:HAc (79: 20:1, *v*/*v*/*v*) for 90 min using a VWR DVX-2500 shaker (VMR International Ltd., Leicestershire, UK). After extraction, the extract of the unspiked samples was spiked and 500 µL of the extract was diluted with 500 µL of the diluent ACN:H_2_O:HAc (20:79:1, *v*/*v*/*v*). Five µL of each diluted extract was injected twice into the LC-MS/MS system for analysis. A total of 500 µL of the working standard solution was diluted with ACN:H_2_O (50:50, *v*/*v*) to achieve the desired concentration of each analyte in the working standard. Quantification was performed via external calibration using a nine-point calibration curve achieved by serial dilutions of the multi-analyte working standard solution. Data were further processed using Analyst^®^ 1.7.1 and SCIEX OS-Q 3.0.

The apparent recovery (RA), matrix effect (SSE), and extraction efficiency (RE) were calculated using Equations (3)–(5) below.

(3) $$RA=\frac{area\;of\;sample\;spiked\;before\;extraction}{area\;of\;neat\;solvent\;standard}\times 100$$

(4) $$SSE=\frac{area\;of\;sample\;spiked\;after\;extraction}{area\;of\;neat\;solvent\;standard}\times 100$$

(5) $$RE=\frac{RA}{SSE}\times 100$$

#### 4.5.4. Sample Preparation and Extraction

The initial mycotoxin concentrations of the oat grains were analysed. Following the storage experiment, 120 contaminated oat samples (including replicates) were analysed for mycotoxins. The oats were ground with a high-power 1000 W blender (Geroge, Watford, UK). A volume of 20 mL of extraction solvent (ACN:H_2_O:HAc, 79:20:1, *v*/*v*/*v*) was added to 5 g of ground oat grains. Extraction was carried out for 90 min using a multitube vortex (VWR DVX-2500, VMR International Ltd., Leicestershire, UK), followed by centrifugation for 15 min at 5000 rpm on a Rotina 380R centrifuge (Hettich, Tuttlingen, Germany Zentrifuge). Then, 500 µL of the extract was diluted with 500 µL of dilution solvent (ACN/H_2_O/HAc, 20:79:1, *v*/*v*/*v*). A total of 5 µL of the diluted extract was injected into the LC-MS/MS system for analysis.

### 4.6. Statistical Analyses

The datasets were analysed with Statistica 14.0.1, JMP^®^ Pro 17, and RStudio 2024.04 in R 4.4.1. The normal distribution of data was examined with the normal plots of the residuals. When data failed the normality test, they were transformed to square root or logarithm values to achieve normality. Transformed data were normally distributed; therefore, factorial ANOVA was used to analyse the effect of the interactions between the storage conditions on respiration rates and mycotoxin concentrations. The Tukey Honest Significant Difference (HSD) post hoc test was used to evaluate differences among means for the interactions of storage conditions. The non-parametric comparison for each pair (Wilcoxon method) evaluates significant differences in fungal populations and mycotoxin concentrations for each storage condition. Statistical analyses performed were considered significant when *p*-values were <0.05. The concentrations of mycotoxins below the limit of detection and quantitation were assigned with values of LOD/2 and LOQ/2, respectively, for statistical analysis [34].

## Figures and Tables

**Table 1:** Mean values (*n* = 9 ± SD) of *Fusarium* and *Penicillium* populations (log_10_ CFUs/g) in naturally contaminated oats at all storage conditions.

Fungal Genera	T (°C)	Days	0.70 a_w_		0.90 a_w_		0.95 a_w_	
			MEA+	DG18+	MEA+	DG18+	MEA+	DG18+
*Fusarium*	15	10	-	-	-	2.9 ± 2.7	-	-
		20	3.3 ± 3.4	2.1 ± 2.3	3.3 ± 3.3	2.7 ± 2.6	-	-
	20	10	-	2.8 ± 2.8	2.1 ± 2.3	2.6 ± 2.5	6.2 ± 6.4	-
		20	4.1 ± 4.0	-	-	-	-	-
*Penicillium*	15	10	3.1 ± 2.9	-	2.9 ± 3.1	2.8 ± 2.7	6.7 ± 6.2	6.8 ± 0.0
		20	2.9 ± 0.0	2.1 ± 2.3	3.3 ± 3.4	2.9 ± 3	7.0 ± 6.6	6.9 ± 6.4
	20	10	3.5 ± 3.4	3.6 ± 3.5	2.9 ± 3.0	3.2 ± 3.1	6.9 ± 6.2	6.9 ± 6.1
		20	3.1 ± 2.8	2.9 ± 2.6	5.3 ± 5.2	5.6 ± 0.0	7.0 ± 6.9	7.0 ± 6.9

SD: standard deviation. “-” represents no growth. T: temperature. a_w_: water activity. DG18+: Dichloran Glycerol 18 agar + Chloramphenicol. MEA+: Malt Extract Agar + Chloramphenicol. An amount of 0.70 a_w_ was used as the control condition. Non-parametric comparison for each pair using the Wilcoxon method shows no significant differences in the fungal populations among the three a_w_ levels for each media for each day and each temperature (within each row).

**Table 2:** Frequency of isolation (%) of *Fusarium*, *Penicillium*, and *Aspergillus* in naturally contaminated oats at all storage conditions.

Fungal Genera	T (°C)	Days	0.70 a_w_		0.90 a_w_		0.95 a_w_	
			MEA+	DG18+	MEA+	DG18+	MEA+	DG18+
*Fusarium*	15	10	7	45	12	24	-	-
		20	4	43	15	20	11	-
	20	10	-	49	-	39	11	-
		20	-	49	-	64	1	-
*Penicillium*	15	10	85	96	83	96	100	100
		20	77	87	81	91	100	100
	20	10	79	99	81	97	89	100
		20	72	97	69	97	68	96
*Aspergillus.* sect. *Nigri*	15	10	-	3	-	-	-	-
		20	3	1	-	-	-	-
	20	10	-	-	-	1	17	9
		20	3	-	-	1	33	29
*Aspergillus.* sect. *Flavi*	15	10	-	-	-	-	-	-
		20	-	-	-	-	-	-
	20	10	-	-	-	-	51	29
		20	-	-	-	-	73	71

“-” represents no growth. T: temperature. a_w_: water activity. DG18+: Dichloran Glycerol 18 agar + Chloramphenicol. MEA+: Malt Extract Agar + Chloramphenicol.

**Table 3:** Mean values (n = 3 ± SD) of respiration rates (µg CO_2_ kg^−1^ h^−1^) in naturally contaminated oats.

	T °C	Days	0.70 a_w_	0.90 a_w_	0.95 a_w_
Naturally contaminated oats	15	0	0.4 ± 0.3 ^a^	1.9 ± 0.5 ^b^	8.4 ± 2.3 ^c^
		10	0.2 ± 0.6 ^a^	1.9 ± 0.6 ^b^	34.2 ± 4.0 ^c^
		20	0.2 ± 0.6 ^a^	2.1 ± 1.9 ^b^	29.8 ± 0.1 ^c^
	20	0	1.3 ± 0.4 ^a^	10.8 ± 1.0 ^b^	11.7 ± 2.3 ^b^
		10	2.0 ± 1.0 ^a^	63.0 ± 11.7 ^b^	133.0 ± 0.0 ^c^
		20	3.4 ± 2.6 ^a^	48.0 ± 11.1 ^b^	111.2 ± 28.4 ^c^

SD—standard deviation. T—temperature. a_w_—water activity. Different letters show significant differences in the RRs for each aw and days in each row.

**Table 4:** Mean values (n = 48) of the inter-day precision within-lab reproducibility (WLR) method validation for oat grains.

Components	R_A_ (%)	RSD (%)	R_E_ (%)	SSE (%)	LOD (µg/kg)	LOQ (µg/kg)
HT-2 toxin	88	7	92	96	4.1	13.6
HT-2-glucoside	97	6	104	93	3.9	13.2
T-2 toxin	81	9	79	102	3.7	12.4
Ochratoxin A	97	17	105	92	0.9	2.9

R_A_—apparent recovery, R_E_—extraction efficiency, RSD—relative standard deviation, SSE—signal suppression/enhancement, LOD—limit of detection, LOQ—limit of quantitation.

**Table 5:** The mean values (n = 4 ± SD) of the mycotoxin concentrations in the naturally contaminated oats.

Mycotoxins (µg/kg)							
				Ochratoxin	Type A Trichothecenes		
Treatment	T (°C)	Days	a_w_	OTA	HT-2-Glc	HT-2	T-2
Naturally contaminated oats	15	10	0.7	<LOQ *	86.4 ± 41.8 ^a^	68.4 ± 34.6 ^a^	71.3 ± 67.1 ^a^
			0.9	<LOQ *	73.0 ± 24.7 ^a^	134 ± 35 ^b^	41.1 ± 22.7 ^c^
			0.95	17.1 ± 16.8 *	67.2 ± 14.5 ^a^	231 ± 154 ^a^	16.9 ± 16.8 ^b^
		20	0.7	<LOQ *	61.5 ± 6.5 ^a^	142 ± 93 ^a^	62.3 ± 47.3 ^a^
			0.9	24.5 ± 31.2 *	60.0 ± 14.8 ^a^	102 ± 73 ^a^	44.9 ± 24.8 ^a^
			0.95	187 ± 131 *	44.8 ± 19.2 ^a^	130 ± 72 ^b^	18.6 ± 19.1 ^c^
	20	10	0.7	<LOQ *	62.7 ± 11.3 ^a^	91.6 ± 54.5 ^a^	66.5 ± 9.1 ^a^
			0.9	5.4 ± 4.9 *	43.8 ± 17.9 ^a^	41.3 ± 25.7 ^a^	27.1 ± 23.5 ^a^
			0.95	19.0 ± 19.5 **	47.5 ± 18.4 ^a^	58.0 ± 43.1 ^a^	18.1 ± 16.7 ^b^
		20	0.7	<LOD *	47.9 ± 27.4 ^a^	46.8 ± 21.7 ^a^	224 ± 268 ^a^
			0.9	48.2 ± 65.3 *	46.1 ± 10.6 ^a^	36.7 ± 11.1 ^a^	1281 ± 1335 ^a^
			0.95	86.2 ± 66.7 *	22.8 ± 11.6 ^a^	<LOD ^b^	1471 ± 1131 ^a^

SD—standard deviation. T-2—T-2 toxin. HT-2—HT-2 toxin. HT-2-glucoside. OTA—ochratoxin A. a_w_—water activity. T—temperature. LOD: limit of detection. LOQ: limit of quantitation. Concentrations of analytes <LOD and <LOQ are assigned with values of LOD/2 and LOQ/2 [31], respectively. Using non-parametric comparison for each pair (Wilcoxon method), different * show significant differences in OTA concentrations among a_w_ for each day and each temperature (within the column). The same letters show no significant differences among the type A trichothecenes concentrations at each a_w_, each day, and each temperature (within each row).

**Table 6:** Pearson correlation coefficient (r) and *p*-values of respiration rates and mycotoxin concentrations in contaminated oats.

Variable	Correlations. Marked Correlations Are Significant at *p* < 0.05000 N = 60						
	CO_2_	OTA	HT-2 Toxin	T-2 Toxin	HT-2-Glc	Sum T2HT2HT2GLc	Sum T2HT-2
CO_2_	1.0000	0.5322	−0.0916	−0.1931	−0.2902	0.0039	−0.0192
	*p* = ---	*p* = 0.000	*p* = 0.486	*p* = 0.139	*p* = 0.025	*p* = 0.976	*p* = 0.884
OTA	0.5322	1.0000	−0.0917	−0.0763	−0.2946	−0.0009	0.0483
	*p* = 0.000	*p* = ---	*p* = 0.486	*p* = 0.562	*p* = 0.022	*p* = 0.995	*p* = 0.714
HT-2 toxin	−0.0916	−0.0917	1.0000	−0.1646	0.6274	0.0181	0.1194
	*p* = 0.486	*p* = 0.486	*p* = ---	*p* = 0.209	*p* = 0.000	*p* = 0.891	*p* = 0.364
T-2 toxin	−0.1931	−0.0763	−0.1646	1.0000	−0.1716	0.8776	0.8708
	*p* = 0.139	*p* = 0.562	*p* = 0.209	*p* = ---	*p* = 0.190	*p* = 0.00	*p* = 0.00
HT-2-Glc	−0.2902	−0.2946	0.6274	−0.1716	1.0000	−0.0807	−0.0963
	*p* = 0.025	*p* = 0.022	*p* = 0.000	*p* = 0.190	*p* = ---	*p* = 0.540	*p* = 0.464
Sum T2HT2HT2Glc	0.0039	−0.0009	0.0181	0.8776	−0.0807	1.0000	0.9799
	*p* = 0.976	*p* = 0.995	*p* = 0.891	*p* = 0.00	*p* = 0.540	*p* = ---	*p* = 0.00
Sum t2ht2	−0.0192	0.0483	0.1194	0.8708	−0.0963	0.9799	1.0000
	*p* = 0.884	*p* = 0.714	*p* = 0.364	*p* = 0.00	*p* = 0.464	*p* = 0.00	*p* = ---

**Table 7:** Optimised MS/MS parameters for the analysed mycotoxins, including precursor ions, product ions, declustering potential (DP), collision energy (CE), and collision cell exit potential (CXP).

Mycotoxins	Precursor Ion (*m*/*z*)	Product Ion (*m*/*z*)	DP (V)	CE (V)	CXP (V)
T-2 toxin	484.3	215.2	57	29	17
	484.3	185.1	27	33	11
HT-2 toxin	447.4	345.1	131	27	20
	442.2	323.2	50	15	16
HT-2-Glucoside	604.3	323.1	101	17	16
	604.3	263.1	101	23	14
Ochratoxin A	404.0	239.0	91	37	16
	404.0	102.0	102	105	14

## Data Availability

The original contributions presented in this study are included in the article/Supplementary Material. Further inquiries can be directed to the corresponding author.

## Supplementary Materials

The following supporting information can be downloaded at: https://www.mdpi.com/article/10.3390/toxins17030132/s1, Figure S1: Pictorial representation of fungal populations/isolations in contaminated oats on MEA+ media at all water activity (a_w_) levels at 15 °C for direct plating (DP) and serial dilution (SD) methods; Figure S2: Mean values of CO_2_ respiration rates in contaminated oats at 15 and 20 °C for 20 days with standard error bars (n = 3). Bars represent respiration rates at each storage day (day 0, day 10, and day 20). * shows significant differences between each water activity level at each temperature using the Wilcoxon test (*p* < 0.05). a_w_—water activity; Figure S3: Plot of the predicted against the known values of T-2 + HT-2 with residuals and normal Q-Q plots.; Figure S4: Plot of the predicted against the known values of ochratoxin A with residuals and normal Q-Q plots; Table S1: Pearson correlation coefficient (r) and *p*-values of respiration rates and mycotoxin concentrations in contaminated oats; Figure S5: Moisture adsorption curves for oat grains.
