# Supplementary material for: Real-Time CO2 Production Monitoring in Stored Oats as an Indicator of Type A Trichothecenes and Ochratoxin A Contamination Under Simulated Environmental Conditions
Source: Toxins (Basel). 2025 Mar 11;17(3):132. doi: 10.3390/toxins17030132 (PMC11945852; doi:10.3390/toxins17030132)
Supplement: Supplementary file 1 [file toxins-17-00132-s001.zip › toxins-3466772-supplementary.pdf]

## Supplementary materials

# Real-time CO2 production monitoring in stored oats as an indicator of type A trichothecenes and ochratoxin A contamination under simulated environmental conditions.

**Visual representations of fungal contamination in stored oats.**

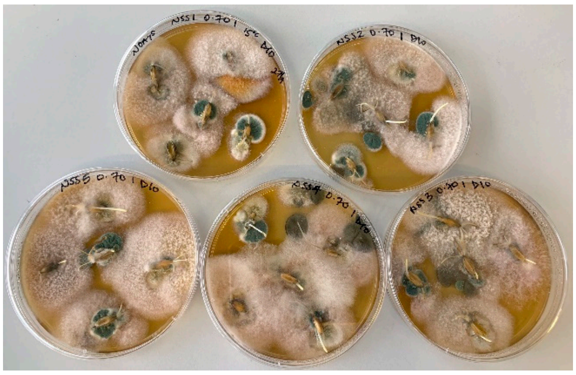

NO, 0.70$a_w$ and 15°C, DP

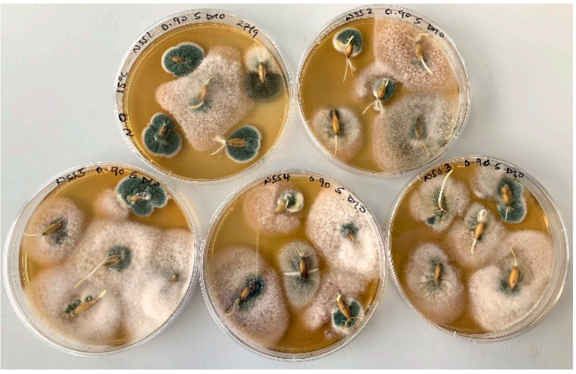

NO, 0.90$a_w$ and 15°C, DP

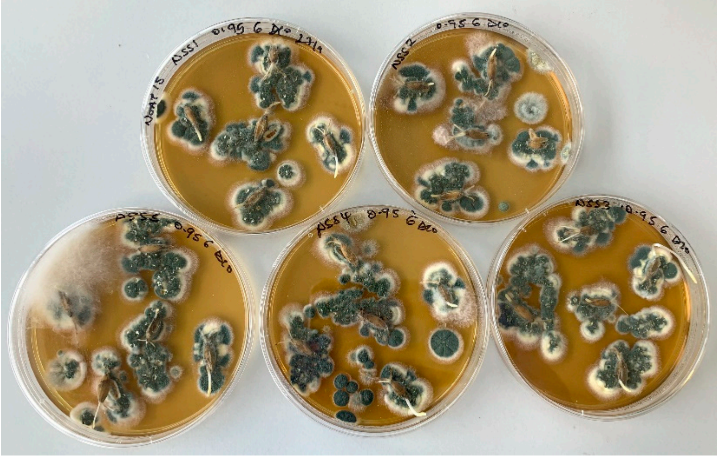

NO, 0.95$a_w$ and 15°C, DP

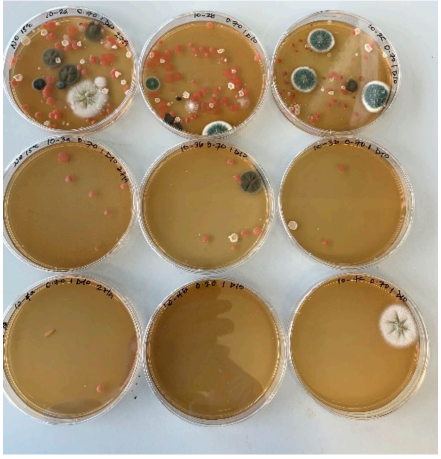

NO, 0.70$a_w$ and 15°C, SD

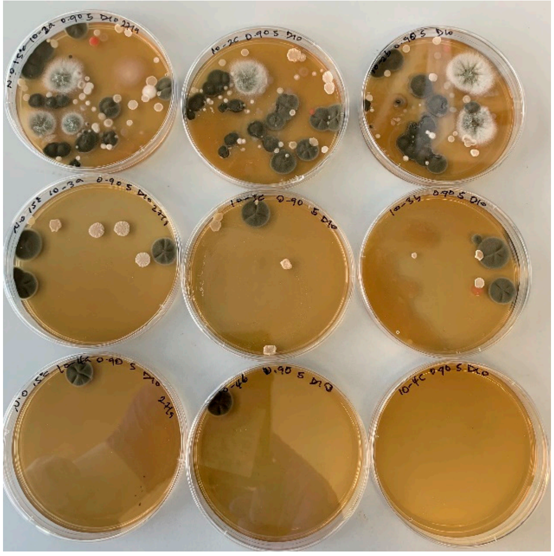

NO, 0.90$a_w$ and 15°C, SD

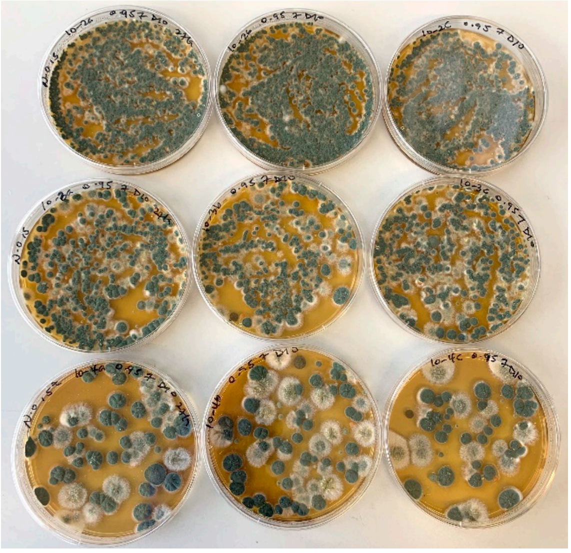

NO, 0.95$a_w$ and 15°C, SD

Figure S1: Pictorial representation of fungal populations/isolations in contaminated oats on MEA+ media at all water activity ($a_w$) levels at 15°C for Direct plating (DP) and Serial dilution (SD) methods.

## Respiration rates produced in stored oats at all storage conditions

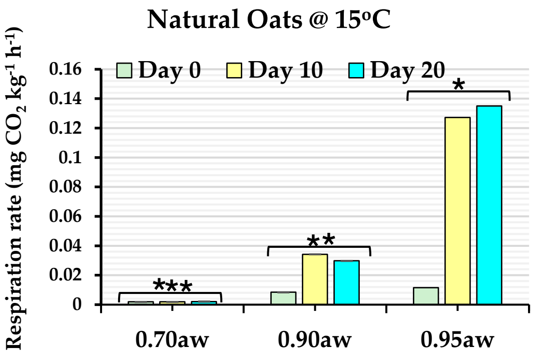


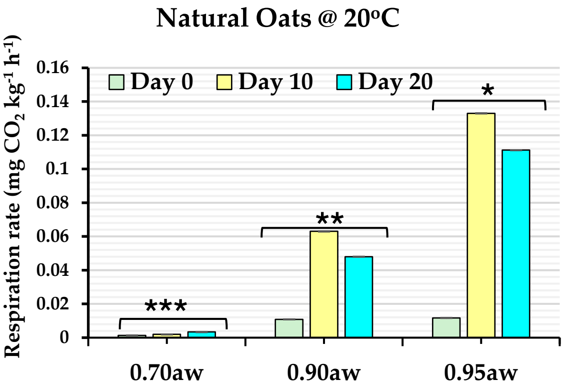


Figure S2: Mean values of $CO_2$ respiration rates in contaminated oats at 15 and 20 °C for 20 days with standard error bars (n=3). Bars represent respiration rates at each storage day (day 0, day 10, and day 20) * shows significant differences between each water activity level at each temperature using the Wilcoxon test ($p < 0.05$). $a_w$- water activity.

## Cross-validation and residual plots of the linear regression model for mycotoxins in oats

The sum of type A trichothecenes

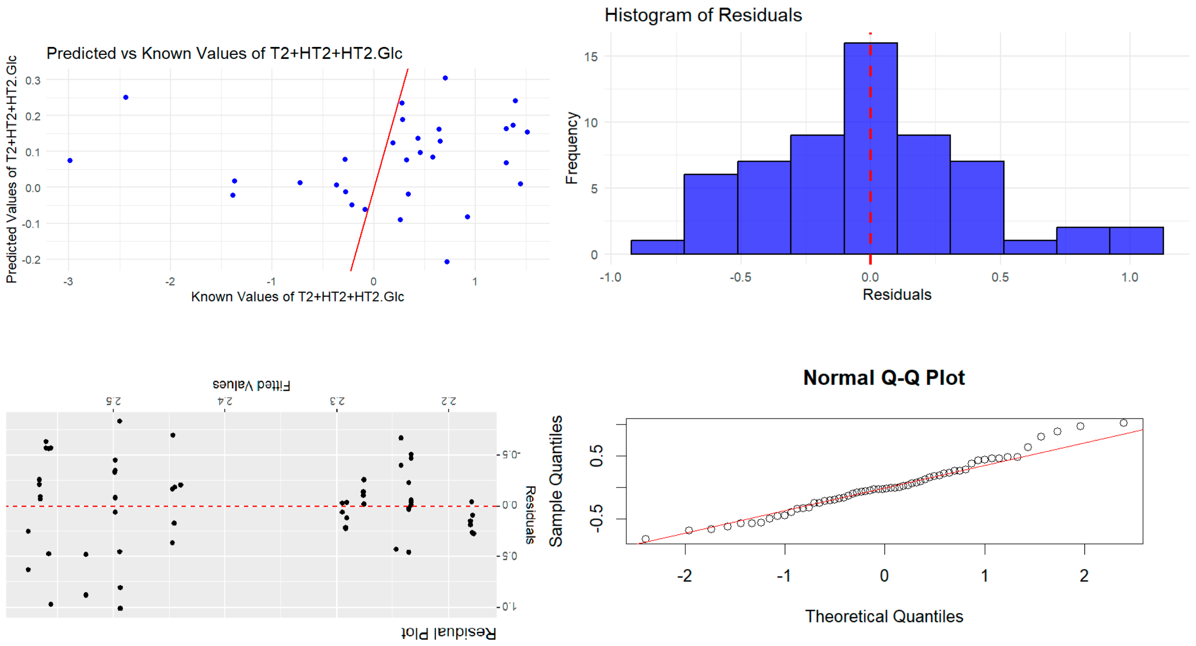


Figure S3: Plot of the predicted against the known values of T-2+HT-2 with residuals and normal Q-Q plots

Ochratoxin A

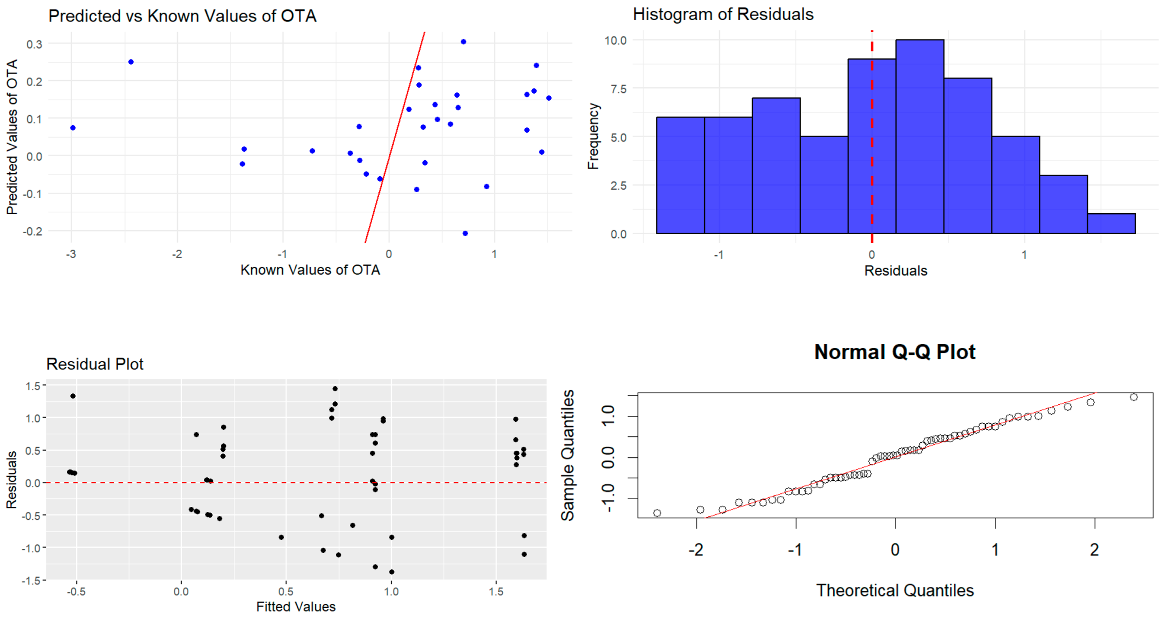


Figure S4: Plot of the predicted against the known values of ochratoxin A with residuals and normal Q-Q plots

Table S1: Pearson Correlation coefficient (r) and p values of respiration rates and mycotoxin concentrations in contaminated oats.

| Variable | Correlations. Marked correlations are significant at p < .05000 N=60 | | | | | | |
|---|---|---|---|---|---|---|---|
| | sqrt $CO_2$ | OTA | HT-2 Toxin | T-2 toxin | HT-2-Glc | Sum T2HT2HT2GLc | Sum T2HT-2 |
| sqrt $CO_2$ | 1.0000 | .5322 | -.0916 | -.1931 | -.2902 | .0039 | -.0192 |
| | p= --- | p=.000 | p=.486 | p=.139 | p=.025 | p=.976 | p=.884 |
| LN OTA | .5322 | 1.0000 | -.0917 | -.0763 | -.2946 | -.0009 | .0483 |
| | p=.000 | p= --- | p=.486 | p=.562 | p=.022 | p=.995 | p=.714 |
| LN HT-2 Toxin | -.0916 | -.0917 | 1.0000 | -.1646 | .6274 | .0181 | .1194 |
| | p=.486 | p=.486 | p= --- | p=.209 | p=.000 | p=.891 | p=.364 |
| LN T-2 toxin | -.1931 | -.0763 | -.1646 | 1.0000 | -.1716 | .8776 | .8708 |
| | p=.139 | p=.562 | p=.209 | p= --- | p=.190 | p=0.00 | p=0.00 |
| LN HT-2-Glc | -.2902 | -.2946 | .6274 | -.1716 | 1.0000 | -.0807 | -.0963 |
| | p=.025 | p=.022 | p=.000 | p=.190 | p= --- | p=.540 | p=.464 |
| LN Sum T2HT2HT2Glc | .0039 | -.0009 | .0181 | .8776 | -.0807 | 1.0000 | .9799 |
| | p=.976 | p=.995 | p=.891 | p=0.00 | p=.540 | p= --- | p=0.00 |
| LN Sum t2ht2 | -.0192 | .0483 | .1194 | .8708 | -.0963 | .9799 | 1.0000 |
| | p=.884 | p=.714 | p=.364 | p=0.00 | p=.464 | p=0.00 | p= --- |

**Moisture Adsorption curve for oat grains.**

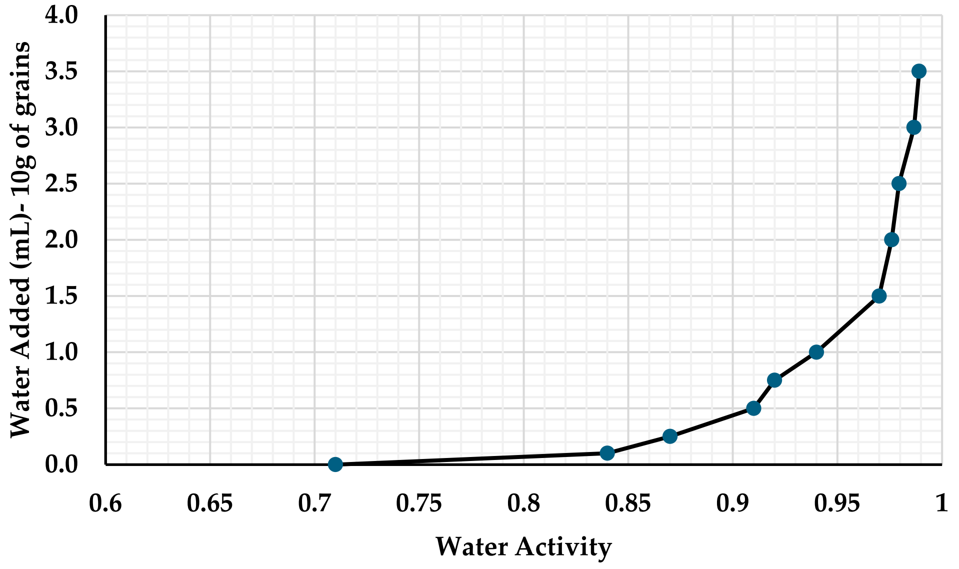


**Figure S5: Moisture Adsorption Curves for oat grains.**
